# Cytoskeletal anchorage of different Dsg3 pools revealed by combination of hybrid STED/SMFS-AFM

**DOI:** 10.1007/s00018-022-04681-9

**Published:** 2023-01-05

**Authors:** Michael Fuchs, Mariya Y. Radeva, Volker Spindler, Franziska Vielmuth, Daniela Kugelmann, Jens Waschke

**Affiliations:** 1 Chair of Vegetative Anatomy, Institute of Anatomy, Faculty of Medicine, Ludwig-Maximilians-Universität Munich, Munich, Germany; 2 Department of Biomedicine and Institute of Anatomy, University of Basel, Basel, Switzerland

**Keywords:** Desmosome, Cell adhesion, Desmosomal anchorage, STED, AFM, STED/AFM

## Abstract

Desmoglein 3 (Dsg3) is a desmosomal cadherin mediating cell adhesion within desmosomes and is the antigen of the autoimmune blistering skin disease pemphigus vulgaris. Therefore, understanding of the complex desmosome turnover process is of high biomedical relevance. Recently, super resolution microscopy was used to characterize desmosome composition and turnover. However, studies were limited because adhesion measurements on living cells were not possible in parallel. Before desmosomal cadherins are incorporated into nascent desmosomes, they are not bound to intermediate filaments but were suggested to be associated with the actin cytoskeleton. However, direct proof that adhesion of a pool of desmosomal cadherins is dependent on actin is missing. Here, we applied single-molecule force spectroscopy measurements with the novel single molecule hybrid-technique STED/SMFS-AFM to investigate the cytoskeletal anchorage of Dsg3 on living keratinocytes for the first time. By application of pharmacological agents we discriminated two different Dsg3 pools, only one of which is anchored to actin filaments. We applied the actin polymerization inhibitor Latrunculin B to modify the actin cytoskeleton and the PKCα activator PMA to modulate intermediate filament anchorage. On the cellular surface Dsg3 adhesion was actin-dependent. In contrast, at cell–cell contacts, Dsg3 adhesion was independent from actin but rather is regulated by PKC which is well established to control desmosome turn-over via intermediate filament anchorage. Taken together, using the novel STED/SMFS-AFM technique, we demonstrated the existence of two Dsg3 pools with different cytoskeletal anchorage mechanisms.

**Supplementary Information:**

The online version contains supplementary material available at 10.1007/s00018-022-04681-9.

## Introduction

Atomic force microscopy (AFM) can be applied to perform adhesion measurements between recombinant proteins and cell surface transmembrane proteins. Therefore, AFM topography imaging of cells and subsequent single molecule force spectroscopy (SMFS) measurements are performed. In the imaging-mode, force–distance curves are measured for every pixel with a high imaging velocity resulting in the samples height over a predefined area. Hereafter, SMFS measurements were performed within the exact same area. At each pixel, the cantilever is approached to the surface. During retrace from the surface, adhesion between recombinant and transmembrane proteins can be measured. After each approach and retract cycle, the AFM cantilever is moved to the next position. With the help of this technique, it was possible in the past to gain a deeper understanding of the biological as well as physical properties of desmosomal cadherins in a cellular context [1–5]. The AFM technique revealed two different bond types of cadherin interactions, i.e., bent-bonds and tether-bonds. Both types of interactions can be differentiated by their slopes of the force-distance curve before rupture. Bent-bonds show an increasing negative slope before rupture whereas tether-bonds have a flat slope. This flat slope indicates that the force acting on the AFM cantilever remains constant although the distance between AFM tip and cell surface increases. This is possible because the binding partner molecules inside the plasma membrane are not rigidly anchored to the cytoskeleton but rather is pulled away from the cortex together with the lipids from the surrounding membrane in a force-free manner. In contrast, a bent-bond occurs when the binding partner is properly anchored to the cytoskeleton [6, 7]. Although AFM topography imaging can reveal filamentous structures underneath the cell membrane, the composition of such filaments remains elusive [8–10]. In this work, we used the STED/SMFS-AFM hybrid-technique for the first time for single molecule adhesion measurements on living cells enabling the merge of mechanistic AFM data with protein localization data acquired by stimulated emission depletion (STED) microscopy. This hybrid-technique combines the advantages of both techniques. The super-resolution microscopy technique STED allows for the identification of subcellular structures while in parallel single molecule binding properties, such as binding strength and unbinding position can be examined with the AFM technique. Previous studies have demonstrated that the combination of AFM and super-resolution microscopy is possible on fixed cells [11]. However, application of the technique on living cells remains challenging and has only recently been completed but SMFS adhesion measurements have not been carried out yet [12]. The combination is especially relevant for characterization of adhesion molecules, the molecular binding properties of which are modulated in spatiotemporal manner.

Desmosomes are intercellular junctions required for proper intercellular adhesion, which are abundant in tissues constantly exposed to mechanical stress, e.g., the epidermis. Their function is to establish strong adhesive interactions between adjacent cells and therefore maintain mechanical tissue integrity [13]. The importance of an intact desmosome function can be seen in multiple diseases concerning the heart or the skin, were desmosomes are compromised due to genetic mutations or autoantibodies [14, 15]. Desmosomal adhesion between cells is mediated by desmosomal cadherins, which perform Ca^2+^-dependent homo- and heterophilic interactions also with classical cadherins [16, 17]. Desmosomal cadherins consist of two different subtypes, desmogleins and desmocollins with each having several isoforms (Dsg1–4 and Dsc1–3). Desmosomal cadherins are transmembrane proteins which are anchored via plakoglobin (Pg) and plakophilin isoforms (Pkp1–3) to desmoplakin (Dp) which in turn links the complex to the cytoskeleton [14, 18]. Dsg3 is of special biomedical relevance because it is the autoantigen in the severe blistering skin disease pemphigus vulgaris and it is well established that loss of Dsg3 adhesion significantly contributes to pemphigus vulgaris pathogenesis [19, 20]. Mature desmosomes are attached to the intermediate filament cytoskeleton [14]. In contrast, Dsg3 accumulates outside of the desmosome before being incorporated into the desmosome [21]. It was suggested that this extra-desmosomal Dsg3 pool is linked to the actin cytoskeleton and might resemble an intermediate state of desmosomal cadherins during desmosome assembly [22]. So far, little is known whether adhesion of extra-desmosomal Dsg3 is regulated by actin filaments and where the shift towards intermediate filaments occurs.

Desmosome assembly occurs in several steps. Prior to the desmosome assembly the presence of adherens junctions is required [23]. At first Dsc2-enriched vesicles were found to be transported via kinesin-2 along microtubules to the plasma membrane, a process that is dependent on the presence of Pkp2 [24, 25]. Vesicles containing Dsg2 were thought to arrive later at the plasma membrane, a process depended on kinesin-1 [24]. A more recent study observed Dsg2 clusters first, followed by Dp recruitment and co-clustering with Dsc2 [26]. Subsequently to cadherin accumulation at the plasma membrane, the cytoplasmic plaque needs to be assembled. Depending on E-cadherin (E-Cad), the desmosomal plaque components Pkp2, Pg and Pkp3 are recruited to the plasma membrane. In the first 5 min of cell contact formation, an unattached Dp pool appears at the cell border [27]. Proper translocation of Dp to nascent desmosomes requires proper actin cytoskeleton organization. A Pkp2 complex together with RhoA facilitates actin remodeling [27]. After 15 min, another pool of Dp, connected to the intermediate filament cytoskeleton emerges beneath the plasma membrane [28]. In this pool, Dp is connected via Pkp2 to protein kinase C alpha (PKC $\alpha$) which facilitates efficient movement of Dp along the intermediate filament cytoskeleton due to modulation of interactions between Dp and intermediate filament [29]. Finally, those different Dp precursors move to the cell–cell contact sides to form mature desmosomes. Similar to the different Dp stages during desmosome assembly also two distinct desmosomal cadherin pools exist, one junctional and one non-junctional [25]. Translocation of non-junctional cadherins into the junctional complex is actin-dependent [30]. Further, it is known that Dsg3 laterally clusters outside of desmosomes, prior to desmosome assembly [21]. The junctional pool of cadherins is not soluble in Trition extraction and thus is thought to be anchored to the cytoskeleton compared to the non-junctional pool [31]. Which mechanism facilitates the exchange of the anchorage partner for Dsg3 is not well understood. We therefore addressed the question whether adhesion of extra-desmosomal Dsg3 is regulated by actin filaments.

Therefore, in this study we describe a method to simultaneously characterize the actin organization and probe the single molecule binding properties of a desmosomal cadherin in correlation with the actin cytoskeleton. By application of actin and desmosome modulating pharmacological agents different desmosomal cadherin pools were determined with two distinct anchorage mechanisms. These data demonstrate that STED/SMFS-AFM is helpful to further characterize desmosome turnover.

## Materials and methods

### Cell culture, transfection and reagents

Wildtype murine keratinocytes were isolated and maintained as previously described (kindly provided by Prof. Dr. Hatzfeld, University of Halle (Saale)) [32]. Murine keratinocytes were grown on culture dishes in complete FAD media (ThermoFisher Scientific, Germany). Cells were grown in a low Ca^2+^ medium (0.05 mM CaCl_2_) and switched to a high Ca^2+^ medium (1.8 mM) after reaching confluence to start differentiation. Experiments were conducted 24 h after switching cells to high Ca^2+^ conditions throughout all experiments. For Dsg3 overexpression, cells were grown to 70% confluency and afterwards transiently transfected with pSNAPf-mDsg3-N using Lipofectamin 3000 according to the manufacturer´s instructions as previously described (Invitrogen, Carlsbad, USA) [33]. After 24 h of transfection, cells were switched to high Ca^2+^ medium. The stably keratin5-YFP transfected immortalized human keratinocyte cell line HaCaT (kind gift of Rudolf Leube, RWTH Aachen University) was cultured in Dulbecco´s Modified Eagle Medium which contains 10% FCS (Biochrom, Berlin, Germany), 50 U/ml penicillin (AppliChem, Darmstadt, Germany) and 50 g/ml streptomycin (AppliChem, Darmstadt, Germany) [34]. HaCaT cells were transfected with pSNAPf-mDsg3-N as the murine keratinocytes. 24 h after transfection, cells were used for immunostaining or STED/AFM-SMFS experiments. Latrunculin B (LatB) (Merck, Darmstadt, Germany) was used at a concentration of 2.0 µg/ml for one hour. The PKC inhibitor Bisindolylmaleimide-X (BIM-X) (Enzo Life Sciences, Lörrach, Germany) was used for 1 h at a concentration of 1 µM. The PKCα activator phorbol 12-myristate 13-acetate (PMA) (Merck, Darmstadt, Germany) was applied for 1 h at a concentration of 2.5 µg/ml.

### Electrophoresis and Western blot analysis

Cells were lysed with Triton X-100 buffer (0.5% Triton X-100, 50 mM MES, 25 mM EGTA, 5 mM MgCl_2_, protease inhibitors) to perform protein fractionation. Cell lysates were collected and separated into a Triton—soluble (non-cytoskeletal bound, supernatant) and a Triton—insoluble (cytoskeletal bound, pellet) fraction via centrifugation at 10,000 rpm for 5 min. Protein concentrations were determined using a BCA protein assay kit (Pierce/Thermo Fisher Scientific, Waltham, USA) according to the manufacturers protocol. Western Blotting was conducted as previously described [35]. As primary antibodies we used: Desmoglein 3 pAb (Biozol Diagnostica, Eching, Germany), Desmoplakin pAb (Santa Cruz, Dallas, TX, USA), GAPDH mAb (AviaSysBio, San Diego, CA, USA), Lamin B1 (Santa Cruz, Dallas, TX, USA) and E-Cadherin mAb (BD Transduction, Franklin Lakes, USA). Secondary antibodies were HRP-coupled goat anti rabbit Ab or goat anti mouse Ab (Dianova, Hamburg, Germany).

### Immunostaining, labelling of actin filaments and cell nuclei

Murine keratinocytes were fixed, 24 h after switched to high Ca^2+^ medium, in pure ethanol for 30 min on ice and subsequently 3 min in aceton at − 20 °C temperature. Primary antibodies used for confocal microscopy were Desmoglein 3 pAb (Biozol Diagnostica, Eching, Germany), E-cadherin mAb (BD Transduction, Franklin Lakes, USA). Actin filaments were labelled using Alexa 488-phalloidin (Dianova, Hamburg, Germany) and cell nuclei were stained by DAPI (Roche, Mannheim, Germany). For STED microscopy primary antibodies were Desmoglein 3 pAb (Biozol Diagnostica, Eching, Germany) and Desmoplakin 1 + 2 mAb (Santa Cruz, Dallas, TX, USA). For confocal microscopy, images were taken with a Leica SP5 confocal microscope using a 63 × NA 1.4 PL APO objective controlled by LAS AF software (Leica, Mannheim, Germany).

### Purification of recombinant Dsg3-Fc construct

Purification of recombinant human Dsg3-Fc proteins were done as previously described [36]. Briefly, the used Dsg3-Fc construct contains the full extracellular domain of the Dsg3 isoform. Constructs were expressed in Chinese hamster ovarian cells and isolation of the recombinant protein from the supernatants was performed by usage of a protein-A agarose affinity chromatography (Life Technologies, Carlsbad, CA).

### Sample preparation

Cells were grown to confluency in low Ca^2+^ medium and subsequently switched to high Ca^2+^ (1.8 mM) for 24 h to induce differentiation. Staining of the cells was carried out according to manufacturers’ protocol. Briefly, cells were washed one time with complete high Ca^2+^ medium followed by 1 h incubation with Sir F-Actin in complete high Ca^2+^ media (1 µM) (Spirochrome, Denver, USA). Next, cells were washed one time with medium and placed into the BioCell-holder at a pre-selected temperature of 37 °C (Bruker Nano, Berlin, Germany) within the STED/SMFS-AFM setup.

### Atomic force microscopy (AFM)

AFM measurements within the STED/SMFS-AFM setup were conducted with a NanoWizard 4 AFM (Bruker Nano, Berlin, Germany). The experimental sample preparation was done as thoroughly described in previous publications [17, 37]. In short, recombinant proteins (0.15 mg/ml) were linked via a flexible heterobifunctional benzaldehyde polyethylene glycol (PEG) linker (Broadpharm, San Diego, USA) to a flexible Si_3_N_4_ AFM cantilever (MLCT probes, nominal spring constant 0.03 N/m, tip radius 20 nm; Bruker, Mannheim, Germany). AFM adhesion measurement on living murine keratinocytes were carried out in DMEM medium at a Ca^2+^ concentration of 1.8 mM (Life Technologies). Topography overview images were performed in the Quantitative Imaging (QI) mode to obtain information about the cell topography (Settings: setpoint = 0.3 nN, *Z*-length = 1.5 µm, extend/retract speed = 10 µm/s). For adhesion measurements the Force Mapping mode in the SPM Control v.4 software (JPK Instruments, Berlin, Germany) was applied (Settings: relative set point = 0.3 nN, *Z*-length = 1.5 µm, extend/retract speed = 10 µm/s, resting contact time = 0.1 s). We chose small areas along the cell–cell contact areas (4 × 1.5 µm or 3 × 2 µm, 40 × 15 px or 30 × 20 px respectively) and small quadratic areas above the nucleus (2.5 × 2.5 µm, 20 × 20 px) to gain information about the single molecule binding properties depending on the localization. Analysis of the measured force-distance curves gave information about interaction probability, nature of the bond (bent or tether), unbinding strength of the interaction (binding strength) as well as the retracted distance until bond disruption (unbinding position) [1, 5]. Characterization of bent- or tether-bonds was made in dependence of the slope of the force-distance curve shortly before rupture (bent; slope < −60 µN/m or; tether; slope > −60 µN/m) [6].

### STED microscopy

Fixation and staining of the cells was done as described above. Imaging of the cells was conducted with the Expert line setup from Abberior equipped with a 100 × oil objective using Imspector image acquisition software (Abberior Instruments GmbH, Göttingen, Germany). The STED effect was attained with a 775 nm pulsed laser, at a 30% laser power and a gating value of 800 ps.

### STED/SMFS-AFM hybrid

The original STED microscope stage was replaced by an isolated Life Science stage containing a sample holder for inverted optical microscopes (Bruker Nano, Berlin, Germany). The NanoWizard 4 AFM scan head was mounted above. For tip calibration, an aqueous reservoir was initially placed onto the sample holder. At first, the laser beam was focused onto the back of the cantilever and properly aligned. Calibration of the tip was done by applying the contact-based method within the SPM software. Briefly, the sensitivity of the tip was determined by acquiring a force–distance curve on a hard surface and linear fitting of the repulsive part of the curve. This enables the conversion of measured voltage values into units of length. The conversion into units of force was done with a thermal noise measurement, to calculate the spring constant. In a next step, the living and stained keratinocytes were put onto the stage. Alignment of the tip above a specific location was down following a standard JPK protocol in the SPM software (DirectOverlay™). The idea behind the DirectOverlay is to optically calibrate the AFM scan field onto the optical microscope image. The basic procedure is to move the AFM tip on 9 predefined positions. For the optical microscope we chose a scan area of 30 × 30 µm with 1024 × 1024 pixels. At each position the optical microscope takes an image with identical scan area and pixel size. The AFM software reads those images and recognizes the AFM tip in the optical image. With this approach optical distortions or other image artefacts are automatically compensated within the SPM software. After calibration, STED images were taken followed by AFM topography images. Both images were superimposed in the SPM software and adhesion measurements were performed.

### Image processing and analysis

ImageJ software (NIH, Bethesda, MD, USA) was applied for STED image analysis. AFM images and force–distance curves were analyzed with the JPK data processing software (JPK Instruments). For unbinding position and binding strength values per experiment, the mean of two median values from all unbinding events per region of interest was applied. For image composition and processing the Photoline software (Computerinsel, Bad Gögging, Germany) was used. Densitometric measurements were done with ImageJ software (NIH, Bethesda, MD, USA). Further data shown in here were evaluated and depicted with Microsoft Excel (Microsoft, Redmond, WA, USA), RStudio (Boston, Massachusetts, USA) and Prism Software version 8 (Graph Pad, San Diego, California, USA).

### Statistics

For statistical significance between two groups a two-tailed Student´s T-test was applied. In case of more than two group comparisons, analysis of variance (one-way ANOVA) followed by Bonferroni post hoc test, was performed. Error bars represent standard deviation. Statistical significance was assumed at *p*-values < 0.05.

## Results

### Principle STED/SMFS-AFM setup and application on living murine keratinocytes

**Fig. 1:**
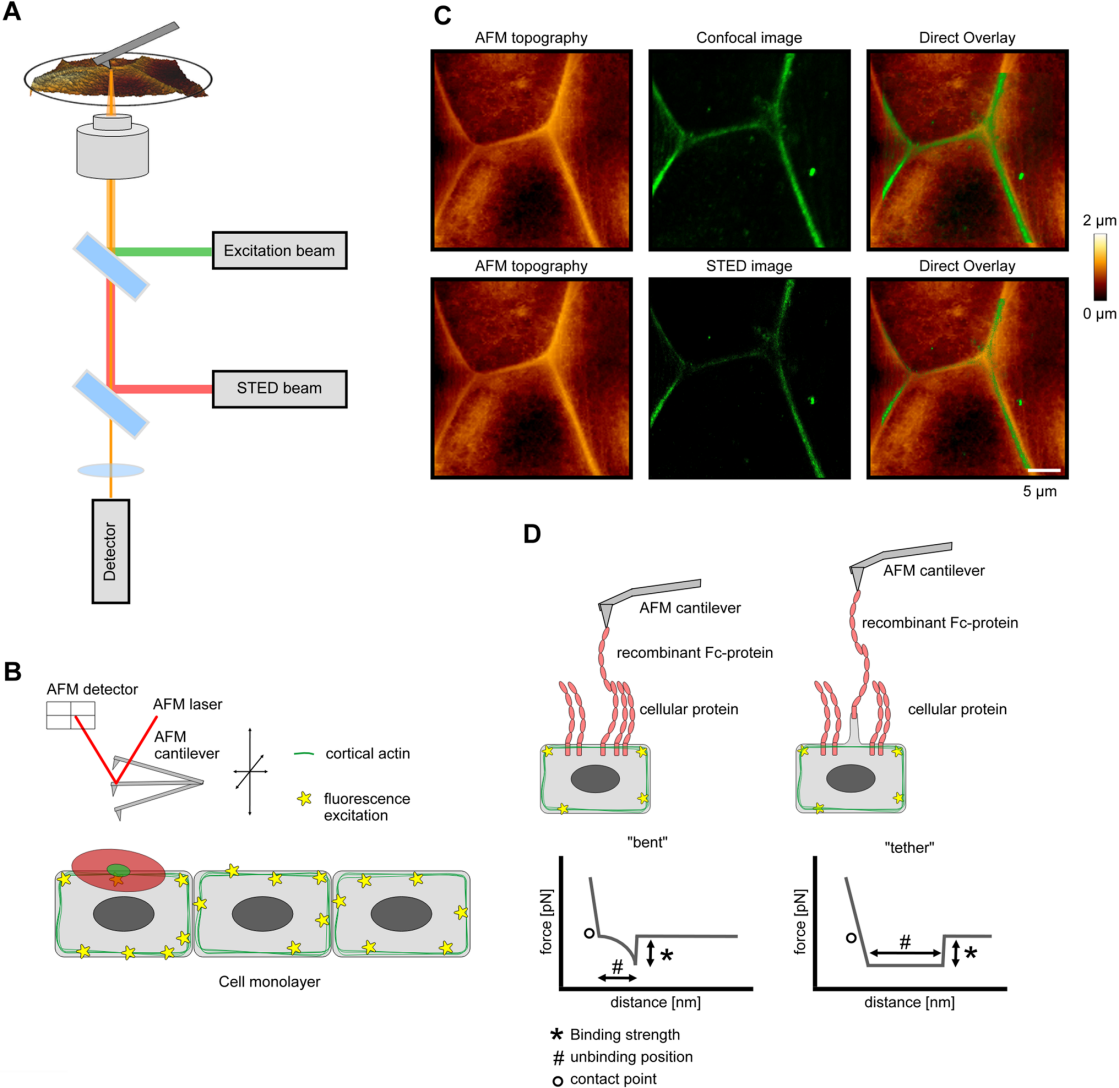
Schematic working principle of the experimental setup. **A** Scheme of a STED/SMFS-AFM system with the main components including the excitation and STED beam, the AFM stage and AFM cantilever as well as the detection unit. The AFM tip can easily be moved over the probe surface. **B** Applied experimental setup in this study. The actin cytoskeleton of living murine keratinocytes is fluorescently labeled, and imaged with the resulting STED laser. Deflection of the AFM cantilever is recorded due to deflection of the AFM-laser. **C** AFM topography image, confocal image and STED image are shown as raw data. Overlay images show the confocal with the AFM image and the STED with the AFM image. **D** Schematic of the two different bond types detected by the AFM, bent- and tether-bonds

The principle working mechanism of the STED/SMFS-AFM setup is shown in Fig. 1A. An excitation beam together with a slightly delayed STED beam is focused on the probe surface. A detector collects the fluorescent signal and an image is constructed. In addition to a conventional STED-Setup, the sample is placed below an AFM scan head. Scanning of the samples surface with the AFM tip results in a topography image. The STED/SMFS-AFM technique has been previously applied on living astrocytes, however adhesion measurements have not been conducted yet [12]. Figure 1B shows the principle working mechanism of the STED/SMFS-AFM setup on living cells carried out in this work. As an example, cells were labeled with the SirActin dye, prior to the STED/SMFS-AFM experiment. Labeled cells were placed in a BioCell holder at 37 °C within the STED/SMFS-AFM setup. After finding the right region of interest, a STED image of the filamentous actin was recorded. In a next step, an AFM topography image was recorded at the same region of interest. Both recorded images were superimposed with the help of the Direct Overlay^®^ calibration tool from JPK instruments (Fig. 1C). In a first step, proof of principle experiments were carried out to demonstrate the feasibility of STED/SMFS-AFM measurements on living murine keratinocytes. In the AFM topography image, cell borders are clearly discernable as elevated ridges in murine keratinocytes compared to their surroundings. This property was exploited in this work to match the AFM topography image with the optical STED image. The improvement of the STED image in contrast to the confocal image can be seen in raw images (Fig. 1C). The Direct Overlay® demonstrates the feasibility of this technique on living murine keratinocytes. With those measurements, we demonstrate that combined STED/SMFS-analyses are possible on living keratinocytes. In keratinocytes, different types of unbinding events exist depending on the anchorage of the desmosomal cadherin [6]. Unbinding events causing the cantilever to bend and abruptly jump back to neutral position are called bent bonds [6]. Contrariwise, initial bending of the cantilever followed by retraction without additional displacement is called tether bond and is usually correlated with higher unbinding positions (Fig. 1D) [38]. The shape of the force-distance curves enables the determination of the binding type as well as binding strength and unbinding position of the interaction event, the latter of which represents the distance between contact point and bond rupture, can be determined (Fig. 1D). The occurrence of tether bonds correlates with poor anchorage of the transmembrane protein to the cytoskeleton and therefore describes an indirect measure for cytoskeletal anchorage [5, 39].

### Actin depolymerization reduces Dsg3 anchorage on cellular surface areas

**Fig. 2:**
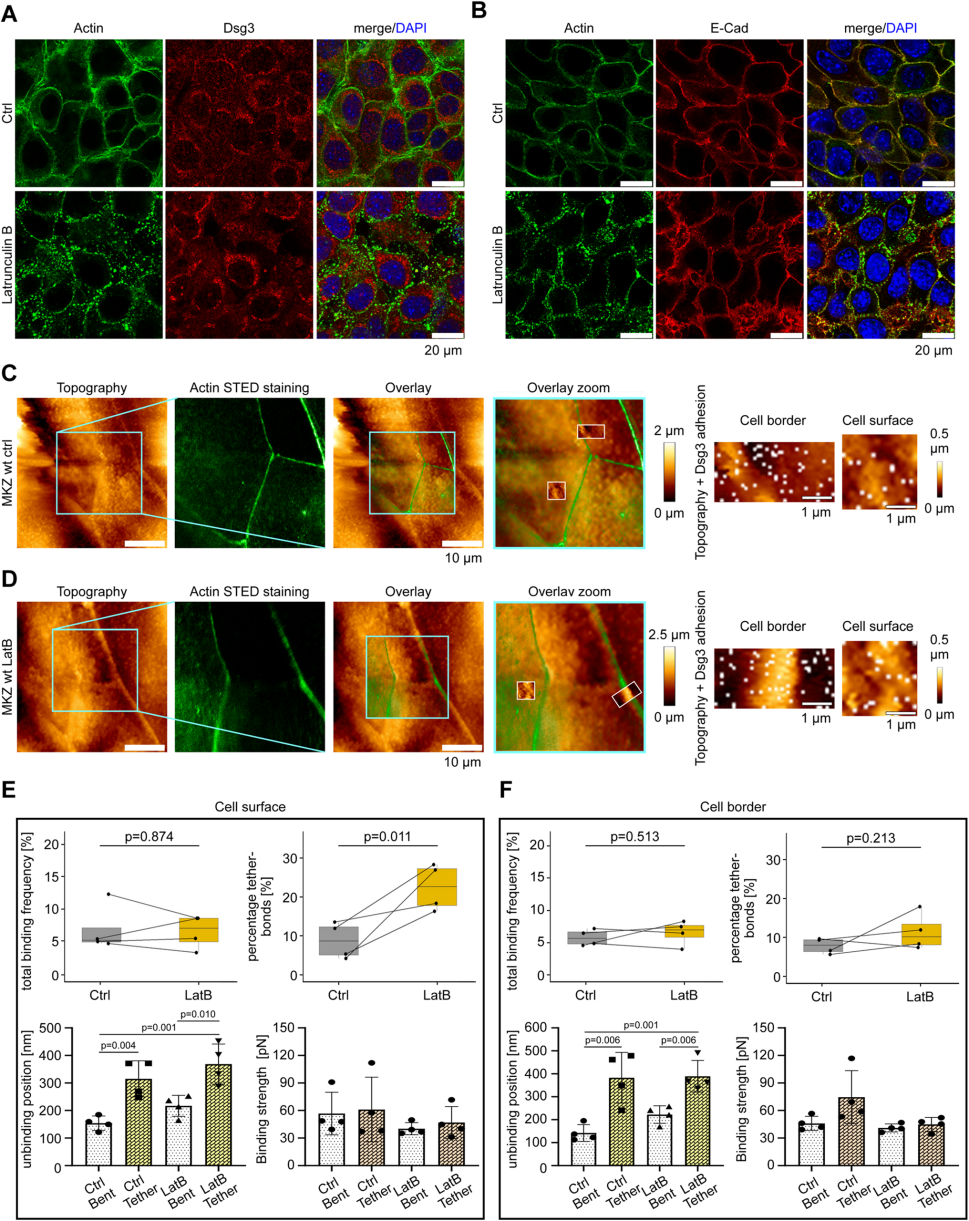
Latrunculin B causes loss of actin anchorage for Dsg3 at the cell surface. **A** Immunostaining of murine keratinocytes with Alexa 488-phalloidin for actin staining and polyclonal antibody for Dsg3. Control cells showed continuous cortical actin and clustered Dsg3 staining. Incubation with LatB led to fragmentation of the actin staining but no significant alterations in Dsg3. **B** Murine keratinocytes were stained with Alexa 488-phalloidin for actin and a monoclonal antibody for E-Cad. Control conditions show E-Cad staining along the cell membrane together with E-Cad. LatB incubation leads to fragmentation of actin staining and more diffusive E-Cad staining. **A, B** Images are representatives of *n* = 3. **C, D** For control and LatB STED/SMFS-AFM measurements, a Dsg3-Fc-functionalized cantilever was used on living murine keratinocytes labeled with SirActin. The same scanning area was used for control and LatB measurements. AFM topography- and STED-image depicts the same cellular area. Overlay zoom images is a zoom into the same dataset. In adhesion maps, white dots represent specific Dsg3 binding-events. **E** Analysis of binding frequency, tether-bond occurrence, unbinding position and binding strength of Dsg3 interactions between control and LatB conditions on the cellular surface. Data points indicate mean values from two different region of interests. **F** Same analysis as done in **E** but parameters were measured at the cell borders. Analysis of *n* = 4 from 4 independent coating procedures, each experiment consists of two different region of interests at the cell surface as well as at the cell border. In every region of interest 600 force distance curves per adhesion maps were measured for cell borders and 625 force distance curves per adhesion maps for cell surface, error bars represent standard deviation of the mean

After successfully establishing the STED/SMFS-AFM technique, we started its application by addressing a desmosome-related biological question. We wanted to investigate the anchorage of different desmosomal cadherin pools accessible with the AFM tip and combine those with STED imaging. Therefore, we modulated the cytoskeletal anchorage of desmosomes by application of pharmacological agents. From previous studies it is known that Dsg3 is associated with actin filaments before being incorporated into the desmosome [22]. Hence, we modulated the actin cytoskeleton with the actin polymerization inhibitor Latrunculin B. Immunostaining and conventional confocal imaging of filamentous actin (F-actin) and Dsg3 after one hour of LatB incubation (2 µg/ml) was performed in a first step (Fig. 2A). Under control conditions F-actin was continuously stained and Dsg3 was present in clusters along cell–cell contacts as well as intracellularly. LatB incubation led to fragmented F-actin staining as expected. The Dsg3 staining was not altered, as shown also by quantification of the integrated density at the cell surface and cell–cell contacts (Fig. S1A). Additionally, we stained for E-Cad which represents the main cadherin of adherens junctions in keratinocytes [40]. Incubation of LatB caused E-Cad to be less linear and more irregular along the cell–cell contacts (Fig. 2B). Dsg3 besides being localized at cell contacts is also present at the free cell surface (Figs. S1C and D). For STED/SMFS-AFM measurements the tip was functionalized with recombinant human Dsg3-Fc comprising the whole extracellular domains of the respective protein as described before [41, 42]. The overall frequency of binding events with a Dsg3-Fc coated tip on keratinocytes in this study was about 5%. Specificity of Dsg3 interactions was confirmed on previous studies on murine keratinocytes via the usage of inhibitory aDsg3 antibodies [10, 37]. Before performing single molecule adhesion measurements on living murine keratinocytes with the STED/SMFS-AFM technique, the overlay from AFM and STED-image was conducted as described above. The elevated cell borders from the AFM topography image were aligned with the corresponding high resolution STED image. Within the STED image the cortical actin structures from neighboring cells are recognizable (Fig. 2C, D). Afterwards, adhesion measurements were conducted in defined regions of interest at cell–cell contact sites and at cell surface areas (Fig. 2C, D). The position of adhesion events were visualized as white pixels (Fig. 2C, D). LatB led to weaker and more diffuse actin staining (Fig. 2D). However, cell–cell contact sites were still visible in AFM topography images indicating that a loss of actin does not profoundly alter cellular topography. For adhesion measurements, we distinguished between two different areas, one directly on the cellular border (cell border: 4 × 1.5 µm) and the other one on the cells surface (cell surface: 2.5 × 2.5 µm). On the cell surface, LatB did not change the binding frequency. However, it increased the proportion of tether vs. bent bonds significantly by 2.2-fold (Fig. 2E). This result indicates that on the cell surface upon LatB treatment the cytoskeletal anchorage Dsg3 proteins was compromised. Additionally, after treatment with LatB the unbinding position was drastically increased for tether bonds compared to bent bonds (Fig. 2E). This indicates that tether bonds show a higher unbinding position. LatB treatment significantly increased the proportion of tether bonds (Figs. 2E and S1E). In contrast, the binding strength was not altered considerably (Fig. 2E). At cell borders, the binding frequency of the Dsg3 interaction as well as the proportion of tether vs bent bonds remained unchanged. The unbinding position between bent and tether bonds was also significantly increased. However, the proportion of tether bonds was not significantly altered after LatB treatment (Figs. 2F and S1E). Similar to measurements on the cell surface, the binding strength remained constant after LatB treatment for both bent and tether bonds (Fig. 2F). The significant increase in tether bonds after LatB treatment on the cell surface can also be displayed by plotting all single unbinding events in one graph (Fig. S1E). Those experiments demonstrate that Dsg3 present on the cellular surface is dependent on the actin cytoskeleton. This is different for Dsg3 molecules at cell borders, where adhesion of Dsg3 appears actin-independent.

### PKC activation disrupts desmosomal cadherin anchorage at cell–cell contacts

**Fig. 3:**
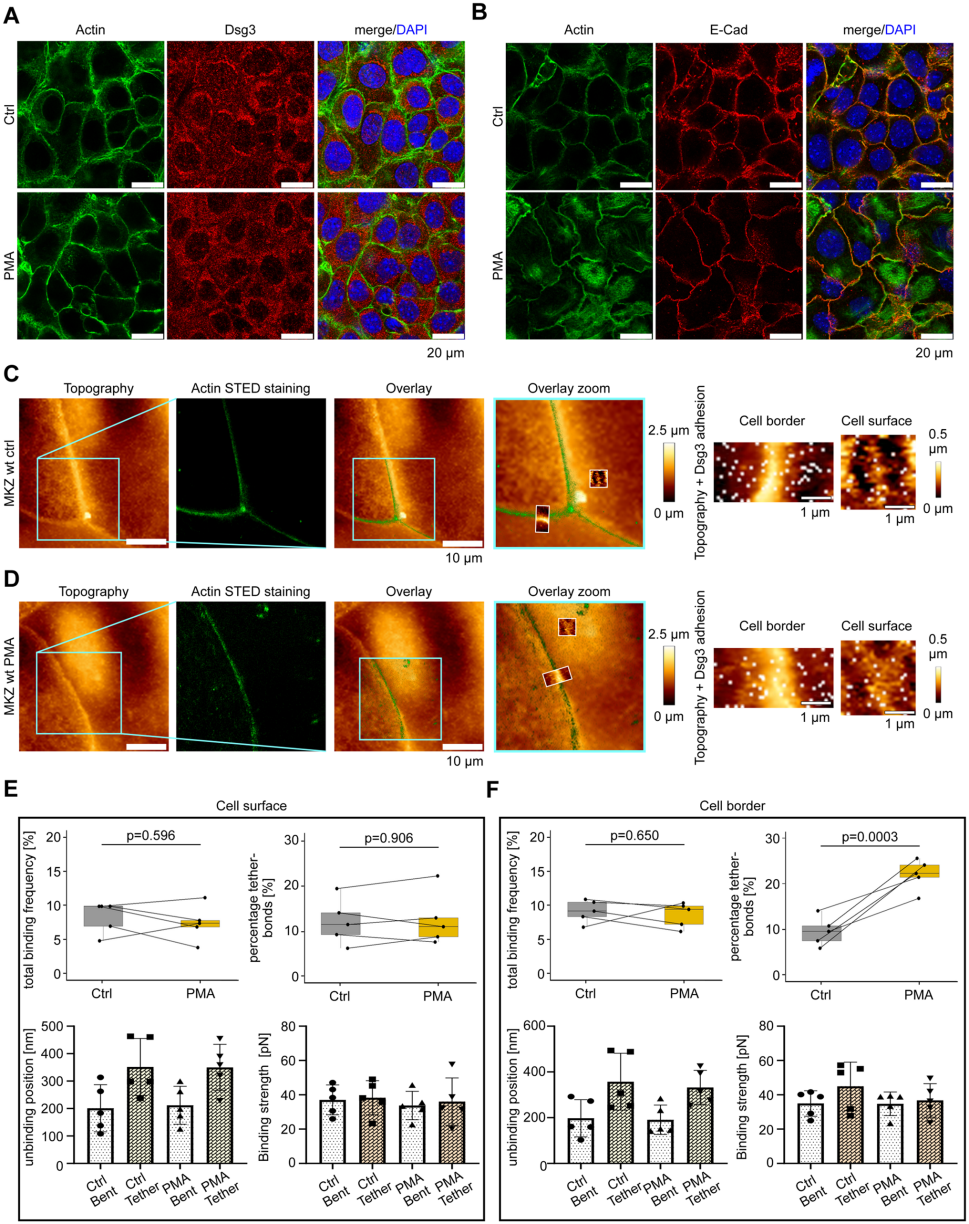
PMA causes loss of cytoskeleton anchorage of Dsg3 molecules at cell–cell contacts. **A**, **B** Murine keratinocytes labelled with Alexa 488-phalloidin for actin staining were immunostained with a polyclonal antibody for Dsg3. PMA treatment showed no difference in protein localization or quantity for Dsg3-, E-Cad- or actin-staining between control or PMA-treated cells. Pictures are representatives of *n* = 3. **C, D** The AFM tip was functionalized with recombinant Dsg3-Fc constructs and was used for topography and adhesion measurements on living murine keratinocytes. For imaging, the same cells were used for control and PMA measurements. AFM topography- and STED-image depicts the same cellular area. Dsg3 binding events are indicated by white dots. **E** Analysis from the cell surface measurements of the binding frequency, proportion of tether bonds, unbinding position and binding strength of Dsg3 interactions between control and PMA treated murine keratinocytes. Data points indicate mean values from two different region of interests. **F** Same analysis of AFM parameters was conducted as done in **E** but measurements were performed at cell–cell borders. Analysis of *n* = 10 from 5 independent coating procedures, each experiment consists of two different region of interests at the cell surface as well as at the cell border. In every region of interest, 600 force distance curves per adhesion maps were measured for cell border and 625 force distance curves per adhesion maps for cell surface, error bars represent standard deviation of the mean

Since anchorage of Dsg3 close to the cell–cell contact sites was not affected by LatB thus appears not to be actin-bound, we asked whether these Dsg3 molecules might be anchored to the intermediate filament cytoskeleton, as it is the case for mature desmosomes. Application of PMA induces PKC$\alpha$-mediated Dp phosphorylation, which in turn leads to architectural changes of the desmosome due to uncoupling from the intermediate filament cytoskeleton [43–45]. Inhibition of PKC$\alpha$ activation leads to hyper-adhesive desmosomes which is associated with stronger desmosomal adhesion [42, 46]. To investigate the impact of PMA on desmosomal cadherin anchorage we started with immunostaining for Dsg3, E-Cad and actin before and after PMA incubation. PMA did not lead to an alteration of actin as well as Dsg3 and E-Cad staining (Fig. 3A, B), which was shown by quantification of the integrated density for Dsg3 staining (Fig. S1B). Dsg3 was still present at the cell surface after PMA treatment (Fig. S1D). In a subsequent step, we again performed STED/SMFS-AFM measurements under presence of PMA. The AFM tip was coated with recombinant Dsg3-Fc. Experiments showed that PMA has no effect on the overall cell topography as well as actin localization (Fig. 3C, D). For Dsg3-Fc adhesion measurements we again compared cell surface and cell borders. On the cell surface the binding frequency of a Dsg3-Fc-coated tip was not significantly altered, and the proportion of bent vs tether bonds was constant before and after PMA treatment. Further, the unbinding position as well as the binding strength did not change due to PMA treatment for adhesion measurements on the cells surface (Fig. 3E). At cell borders, the Dsg3 binding frequency remained constant upon PMA treatment, however, the proportion of tether bonds was significantly increased by about 2.2 fold. Both, unbinding position and binding strength showed no significance dependence on PMA and remained unaltered (Fig. 3F). Thus, comparable to Fig. 2E and F, tether bonds show a higher unbinding position compared to bent bonds, regardless of the treatment. Figure S1F shows all single unbinding events demonstrating a drastic increase in unbinding events of tether bonds after PMA-treatment at cell borders. Control experiments with the PKC inhibitor BIM-X showed no increase in tether bond numbers with a Dsg3-Fc-coated tip. Moreover, we detected a slight decrease of the unbinding position after BIM-X treatment while binding frequency and binding strength were unaltered. Reduced unbinding positions indicate an improved cytoskeletal anchorage of the Dsg3 interaction partners due to PKC inhibition (Figs. S2A and B). We additionally challenged the desmosomal cytoskeletal anchorage via application of PMA and BIM-X at the same time (Fig. S2C). No changes for binding frequency, amount of tether bonds, binding strength as well as unbinding position for a Dsg3-coated tip were detected (Fig. S2D). These results support that PMA-mediated effects were induced via PKC. Taken together, these experiments indicate that Dsg3 at cell contacts is anchored in a PKC-dependent manner and thus most likely via Dp to the intermediate filament cytoskeleton.

### PKC activation disrupts intermediate filament anchorage and triggers desmosome disassembly and internalization

**Fig. 4:**
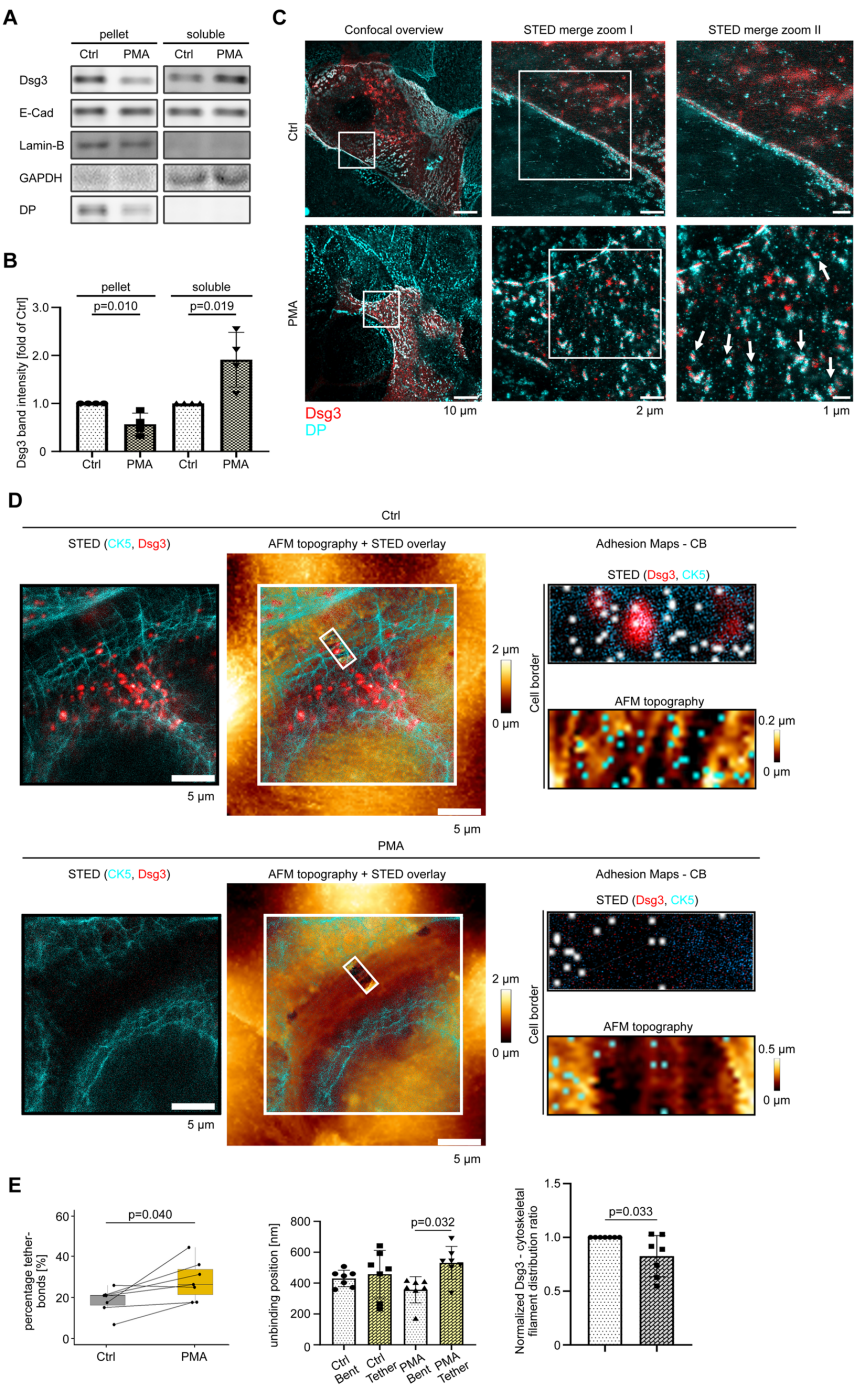
PMA causes internalization of complete desmosomes. **A** Representative Western Blot analysis of the Dsg3 distribution between the soluble and insoluble fraction of a Triton X-100 fraction, comparing control and PMA-treated conditions. **B** Quantification of the Dsg3 band intensity in Trition X-100 soluble and insoluble fraction between control and PMA conditions. *N* = 4, error bars represent standard deviation of the mean. **C** STED microscopy images of murine keratinocytes to visualize the PMA effect on desmosome internalization stained for Dsg3 (red) and Dp (cyan). White arrows represent internalized desmosomes. Pictures are representatives of *n* = 3. **D** Stably transfected CK5-YFP HaCaT cells were transiently transfected with pSNAPf-mDsg3-N. STED-imaging (CK5 in cyan and Dsg3 in red) correlates with AFM topography images. Under control conditions, adhesion events (white dots in STED-image and cyan dots in AFM-topography image) nicely correlate with CK5 filaments or Dsg3 clusters. PMA treatment leads to weakening of CK5 and Dsg3-staining. Pictures are representatives of *n* = 7. **E** Analysis from the cell border measurements between control and PMA treated HaCaT cells of tether bond fraction, unbinding position and Dsg3 binding event distribution ratio between cytoskeleton filament and remaining areas. Data points indicate mean values from two different region of interests. Analysis of duplicates from 7 independent coating procedures, with duplicate 600 force distance curves per adhesion map for cell border and 400 for cell surface per coating procedure, error bars represent standard deviation of the mean

From the previous experiments we concluded that the desmosomal cadherin Dsg3 at cell contact sites may be associated with Dp, which might resemble a nascent desmosome. In this state, Dsg3 is not solely attached to actin filaments but is also partially attached to the intermediate filament cytoskeleton, therefore resembling an intermediate state between assembling and mature desmosomes. Next, we performed triton assays to study cytoskeletal anchorage of Dsg3 molecules biochemically on murine keratinocytes after PMA treatment. The amount of Dsg3 in the insoluble fraction decreased significantly after PMA treatment while it increased significantly in the soluble fraction (Fig. 4A, B). This shift indicates a detachment of Dsg3 molecules from the intermediate filament cytoskeleton upon PKC activation. In comparison, E-Cad distribution was not affected by PMA which is in line with E-Cad being associated with F-actin and independent of the intermediate filament cytoskeleton. Interestingly, PMA decreased the amount of Dp in the insoluble fraction, which suggests the possibility of a triggered desmosome disassembly process (Fig. 4A). To further substantiate the effect of PMA on desmosome uncoupling from the intermediate filament cytoskeleton, we performed STED microscopy imaging. To do so, murine keratinocytes were transiently transfected with SNAP tagged Dsg3 and labeled with SNAP^®^-Cell TMR STAR. Additional immunostaining for Dp was carried out. Under control conditions, we found Dp along cell–cell contacts from two opposing cells with Dsg3 staining in between, revealing a railroad track morphology (Fig. 4C). After PMA treatment, the Dp-Dsg3 sandwich structure appeared strongly fragmented along cell–cell contacts. Furthermore, several internalized and intact desmosomes were detected within the cytosol (white arrows) (Fig. 4C). Additionally, we investigated the PMA effect on the living human keratinocyte HaCaT cell line. Stably transfected CK5-YFP HaCaT cells were transiently transfected with pSNAPf-mDsg3-N. STED images show Dsg3 clusters at cell contact sites as well as extra-desmosomal clusters and keratin filament insertions under control conditions. AFM topography measurements, with a Dsg3-Fc coated tip, at the same location provided topographic information before and after PMA treatment (Fig. 4D). Interaction events within the respective adhesion maps are indicated with white-, in the STED image, and cyan-dots in the AFM topography image. It shows that the localization of adhesion events is preferentially aligned with keratin filaments or directly on the Dsg3 cluster. Remaining adhesion events might originate from endogenous Dsg3 molecules or with unknown cadherin interaction partners. PMA treatment led to a decrease of Dsg3 staining intensity as well reorganization of CK5 filaments, comparable to the effect in murine keratinocytes. Additionally, PMA significantly increased tether bonds and the unbinding positions of tether bonds after PMA treatment at cell borders, both indicate weakened cytoskeletal anchorage (Fig. 4E). Further, display of all data points additionally show the increase in tether bond numbers after PMA treatment (Fig. S3A). The AFM topography and STED images indicate that under control conditions, Dsg3 binding preferentially occurred along cytoskeletal filaments. PMA treatment induced a significant redistribution of these unbinding events away from the cytoskeletal filaments (Fig. 4E). Further AFM parameters were not significantly altered at cell borders and cell surface upon PMA incubation (Figs. S3 B-C). Taken together, those results indicate that PKC activation presumably via Dp phosphorylation results in internalization and disassembly of desmosomes.

## Discussion

In this study, we applied for the first time single molecule AFM force spectroscopy combined with STED super-resolution microscopy on living cells. Thereby, we showed that two distinct pools of Dsg3 molecules exist in murine keratinocytes. One is linked to the actin cytoskeleton on the free cell surface and the other at cell contacts is actin-independent. Both Dsg3 pools may reflect different stages of desmosome assembly. Here the actin-bound pool might resemble extra-desmosomal Dsg3 molecules forming intermediate junctions together with adherens junction components. The actin-independent pool may reflect Dsg3 molecules incorporated into nascent desmosomes. The new hybrid technique enables the visualization of actin fibers and the correlation with single molecule binding properties of desmosomal cadherins.

### Adhesion measurements with the STED/SMFS-AFM hybrid technique

The usage of STED microscopy in parallel with the AFM has a variety of benefits. STED microscopy provides super resolution information about protein distribution and thus enabled the positioning of the AFM tip precisely at a desired region of interest. AFM topography images align to the STED images very accurately, which provides a reliable confirmation of the dataset. AFM images lack the qualitative information from signals within the cell provided by the STED, however it adds mechanistic information about the cells and single molecule binding properties at specific cellular membrane regions. In this study, we showed that the STED/SMFS-AFM technique is applicable on living murine keratinocytes. Previous studies showed the feasibility of STED with AFM on fixed and living cells, however without SMFS adhesion measurements [11, 12]. This correlational approach enables the specific selection of one region due to STED microscopy in sub diffraction regimes and the investigation of this area with the AFM technique.

### Existence of two different anchored Dsg3 pools on the cellular surface

**Fig. 5:**
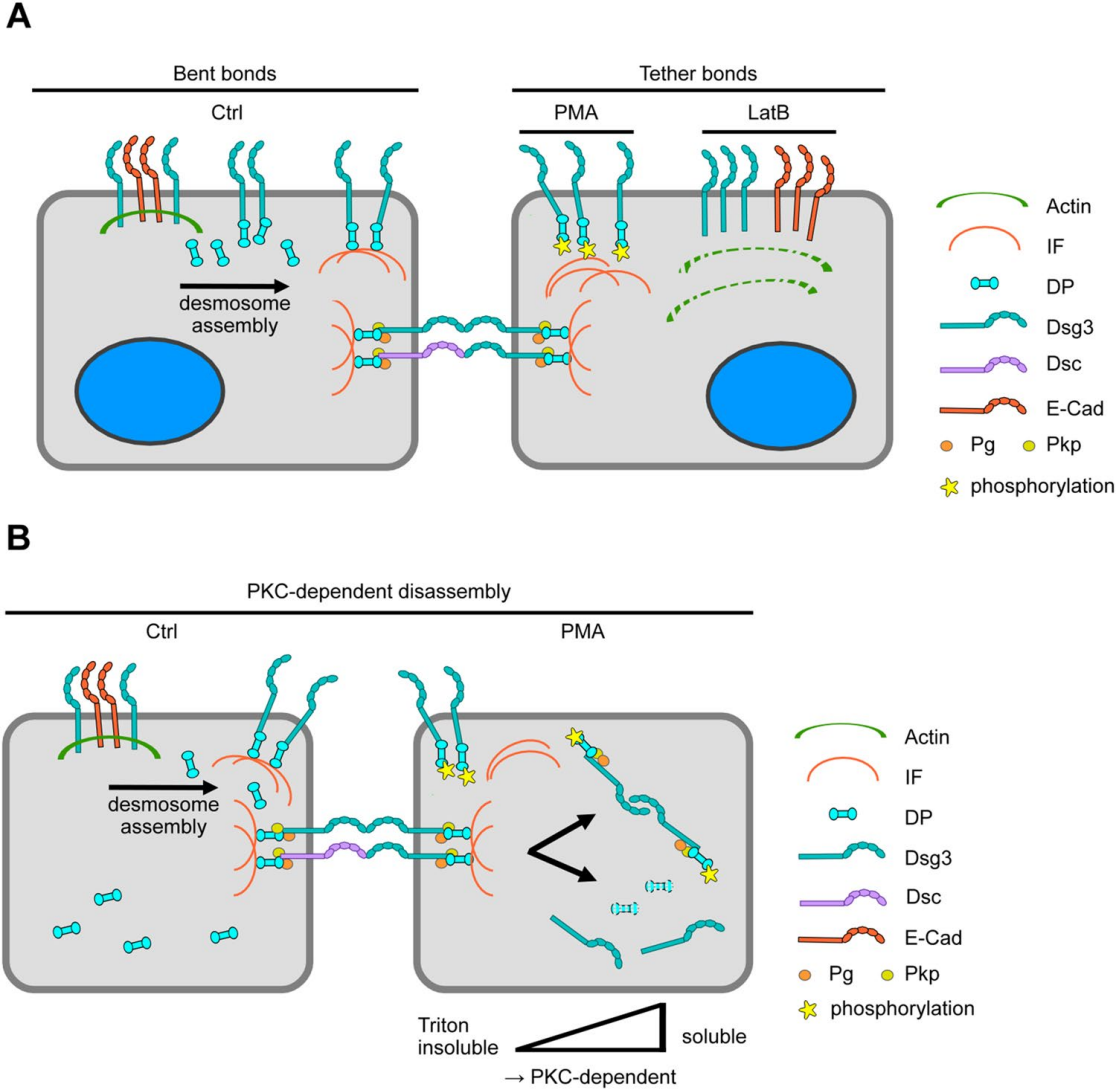
Schematic summary. **A** Schematic summary of the observed desmosome assembly process and the location specific effects of PMA and LatB. **B** Schematic summary of the PMA effect on the detachment of desmosomes form the intermediate filament cytoskeleton and the complete internalization of complete desmosomes

Using this technique together with pharmacological agents, we showed the existence of two different Dsg3 pools in respect to their cytoskeletal anchorage. Previous studies already described two different Dsg pools during the process of desmosome assembly and turnover. During desmosome maturation it is known that desmosomal cadherins rely on microtubule-dependent transport to the plasma membrane [24]. If the arriving desmosomal cadherins are directly incorporated into the desmosome or whether there is a step in between is up for debate. For Dsg3 it is assumed that prior to desmosome incorporation they form clusters within the plasma membrane but outside the desmosome which subsequently laterally incorporate into the desmosome [21]. This is in line with findings that Dsg3 together with E-cadherin forms an intermediate junctions which contains Pg but not Dp [47]. Indeed, for Dsg2 it has been shown that it interacts with the extracellular domain to classical cadherins including E-cad and N-cadherin, which was proposed to facilitate the establishment of nascent desmosomes in epithelial cells as well as of area composite in cardiomyocytes, both on the structural backbone of an adherens junction [16, 17]. Another study suggested that this extra-desmosomal Dsg3 pool is associated with the actin cytoskeleton on the cell surface while the mature desmosomes are anchored to intermediate filaments [14, 22]. Here, we provided evidence for an extra-desmosomal actin-anchored Dsg3 as well as an actin-independent Dsg3-intermediate filament-cytoskeleton pool using the actin-polymerization inhibitor LatB. To test whether the actin-independent Dsg3 pool at cell junctions may be associated with intermediate filament, we applied PMA as previous studies have shown that cytoskeletal anchorage of desmosomal components is PKC-dependent [44]. The single molecule interactions comprised two different kind of bonds, i.e. bent bonds and tether-bonds. Treatment of murine keratinocytes with LatB or PMA led to the formation of tether bonds at the cellular surface or at cell–cell contact sites, respectively. STED/SMFS-AFM setup showed that inhibition of actin polymerization by LatB led to an increase in tether bond frequency at the cell surface, arguing for more actin-bound Dsg3 molecules at this location. In contrast, PMA most likely via PKC-dependent Dp phosphorylation increased the number of Dsg3 tether-bonds at cell–cell contact sites, whereas Dsg3 molecules at the cell surface were unaffected. This experiment demonstrated the existence of two different anchored Dsg3 pools and showed that both pools are accessible with the AFM tip. Moreover, as the Dsg3 pool at cell contacts is susceptible to PMA treatment, this suggests that these cadherin molecules are already in a complex with Dp. We propose that at this localization the mixed junction of Dsg3 together with E-Cad anchored to the actin cytoskeleton may evolve towards nascent desmosomes in a process where actin anchorage is replaced by Dp binding and intermediate filament cytoskeletal anchorage is paralleled by E-cadherin exclusion as was demonstrated before [47, 48] (Fig. 5A). Further, STED/AFM-SMFS experiments with stably transfected CK5 HaCaT cells provided evidence that at cell–cell contact sites, binding events with Dsg3-coated tips preferentially were aligned to intermediate filaments.

Interestingly, we observed that the number of tether bonds correlated with increased unbinding positions for actin-dependent interactions, only. In contrast, for PKC-dependent tether bonds the unbinding position remained unchanged, although the rate of tether bonds roughly doubled. A possible explanation is that Dsg3 at cell contacts is coupled to Dp and possibly other plaque proteins such as PKP which can be deciphered by the absence of a significant increase of unbinding positions. Therefore, we propose that by combined assessment of unbinding position and the rate of tether bonds, it is possible to discriminate adhesion molecules coupled to actin filaments or to desmosomal plaque components.

### PKC activation leads to desmosome internalization

We showed that PKCα activation leads to a detachment of the cell contact-associated Dsg3 pools from the cytoskeleton. It is known that PKCα activation leads to increased phosphorylation of Dp at Ser2849 which modulates its association to intermediate filaments [25, 28]. This is an important mechanism by which PKCα regulates desmosomal disassembly for example upon wounding to increase cell mobility [46, 49]. Further, it is reported that PKCα has a role in regulating the mobility and turnover rate of the desmosomal complex in wound re-epithelialization [50]. In addition, we observed a decrease of both Dsg3 and Dp from the Triton x-100 insoluble pool after PKCα activation. Interestingly, Dsg3 increased in the soluble pool whereas Dp did not shift to the soluble fraction but rather was degraded. During the same time course, super-resolution STED microscopy showed that Dsg3 together with Dp shifted to the cytosol. In the cytoplasm, we observed intact desmosomes upon PMA treatment, indicating that complete desmosomes containing two plaques from two neighboring cells get internalized (Fig. 5B). Similarly, internalization of entire desmosomes following growth factor-induced cell scattering was described previously [51]. It is interesting that at least a fraction of internalized desmosome remained intact and is not disassembled right after internalization. Earlier studies found comparable results for internalized half desmosomes, which are also not disassembled but instead are transported to the centrosomal region where they are degraded [52]. Nevertheless, the data from Triton-X protein extraction indicate that in parallel some desmosomes get disassembled which would explain why Dsg3 but not Dp was enhanced in the soluble fraction.

In summary, using the novel STED/SMFS-AFM technique, we demonstrated the existence of two Dsg3 pools with different cytoskeletal anchorage mechanisms which in the future will allow more precise characterization of cell contact dynamics.

### Supplementary Information

Below is the link to the electronic supplementary material.

Supplementary file1 (DOCX 2735 KB)

## Data Availability

The datasets generated and analyzed during the current study are not publicly available. However, they are available from the corresponding author on reasonable request.

## Abbreviations

AFM: Atomic force microscopy
BIM-X: Bisindolylmaleimide-X
Dp: Desmoplakin
Dsc: Desmocollin
Dsg: Desmoglein
E-Cad: E-cadherin
F-actin: Filamentous actin
LatB: Latrunculin B
Pg: Plakoglobin
PKC$\alpha$: Protein kinase C alpha
Pkp: Plakophilin
PMA: Phorbol 12-myristate 13-acetate
SMFS: Single molecule force spectroscopy
STED: Stimulated emission microscopy
