# Supplementary material for: Cytoskeletal anchorage of different Dsg3 pools revealed by combination of hybrid STED/SMFS-AFM
Source: Cell Mol Life Sci. 2023 Jan 5;80(1):25. doi: 10.1007/s00018-022-04681-9 (PMC9816259; doi:10.1007/s00018-022-04681-9)

**Supplements:**

**Cytoskeletal anchorage of different Dsg3 pools revealed by combination of hybrid STED/SMFS- AFM**

Michael Fuchs^1^, Mariya Y. Radeva^1^, Volker Spindler^2^, Franziska Vielmuth^1^, Daniela Kugelmann^1^* and Jens Waschke^1^*

^1^Chair of vegetative Anatomy, Department I, Faculty of Medicine, Ludwig-Maximilians-Universität Munich, Munich, Germany

^2^Department of Biomedicine and Institute of Anatomy, University of Basel, Switzerland

*contributed equally

Correspondence to: Jens Waschke, Jens.Waschke@med.uni-muenchen.de

**Figurelegends**

*Figure S1: Dsg3 intensity and binding events distribution*

**A:** Quantification of Dsg3 staining of Ctrl and LatB-treatment at cell surface and cell-cell contacts area. Each data point represents the mean values of one individual experiment (N=3). **B:** Dsg3 staining was quantified at cell surface and cell-cell contact areas under control and PMA-treated conditions. Each dot represents the mean values of one individual experiment (N=3). **C-D:** Murine keratinocytes were stained with Dsg3 and Alexa 488-phalloidin for actin. Images show representatives of three individual experiments **C:** Murine keratinocytes grow in two layers under cell culture conditions. Dsg3 shows a distribution over the cell surface area, especially at the superficial layer. **D:** Dsg3 localizes at cell contacts as well as at the cell surface area in the superficial layer under Ctrl, LatB- and PMA-treated conditions, depicted through xz-scans.  **E:** AFM-SMFS measurements with a Dsg3-Fc coated tip, shows every measured unbinding event from all conducted experiments. Shown are the parameters unbinding position and binding strength upon LatB treatment at cell surface and cell border areas. **F:** Presentation of all individual unbinding events with a Dsg3-Fc coated AFM tip from all conducted experiments. Shown are the data points of control and PMA-treated conditions at the cell surface and cell border areas.

*Figure S2: BIM-X abolishes PMA effect on Dsg3 cytoskeletal anchorage*

**A:** Murine keratinocytes were labeled with SirActin for actin staining prior to STED/AFM-SMFS measurements with a Dsg3-Fc coated tip. Topography and adhesion measurements were performed under control and BIM-X treated conditions. Same cellular areas were used for both conditions. Dsg3 binding events are indicated by cyan-colored in AFM-topography and pink-colored dots in fluorescence images. **B:** Analysis from cell border as well as cell surface adhesion maps regarding binding frequency, tether bond fraction, binding strength and unbinding position. Analysis of duplicates from 4 independent coating procedures, 600 force distance curves per adhesion map for cell border and 400 force distance curves per cell surface, error bars represent standard deviation of the mean. Each data points represents the mean value of one independent experiment. **C:** STED/AFM-SMFS measurements on murine keratinocytes fluorescently labeled with SirActin for actin staining. AFM topography and adhesion measurements were performed with a Dsg3-Fc coated tip for control and BIM-X/PMA conditions. The same cellular area was used for both conditions. Dsg3 binding events are indicated by cyan-colored in AFM-topography and pink-colored dots in fluorescence images.  **D: :** Analysis from cell border as well as cell surface adhesion maps regarding binding frequency, tether bond fraction, unbinding force and unbinding position. Analysis of duplicates from 4 independent coating procedures, 600 force distance curves per adhesion map for CB and 400 force distance curves per CS, error bars represent standard deviation of the mean. Each data points represents the mean value of one independent experiment.

*Figure S3: AFM parameters from Dsg3 interactions on Dsg3 overexpressing human keratinocytes*

Human keratinocytes stably transfected CK5-YFP and transiently transfect with pSNAPf-mDsg3-N were used for STED/AFM-SMFS measurements. **A:** For the parameter unbinding position at the cell border, all measured unbinding events from all individual experiments are plotted (N=7). Measurements were performed with a Dsg3-Fc coated tip. **B:** Analysis of force distance curves from the cell border show no changes in binding frequency and binding strength upon PMA treatment. **C:** Analysis from cell surface measurements showed no significant alteration for binding frequency, tether bond percentage, binding strength or unbinding position upon PMA treatment. **B-C:** Analysis of duplicates from 7 independent coating procedures, with duplicate 600 force distance curves per adhesion map for CB and 400 for CS per coating procedure, each data point represents the mean value of one individual experiment. Error bars represent standard deviation of the mean.


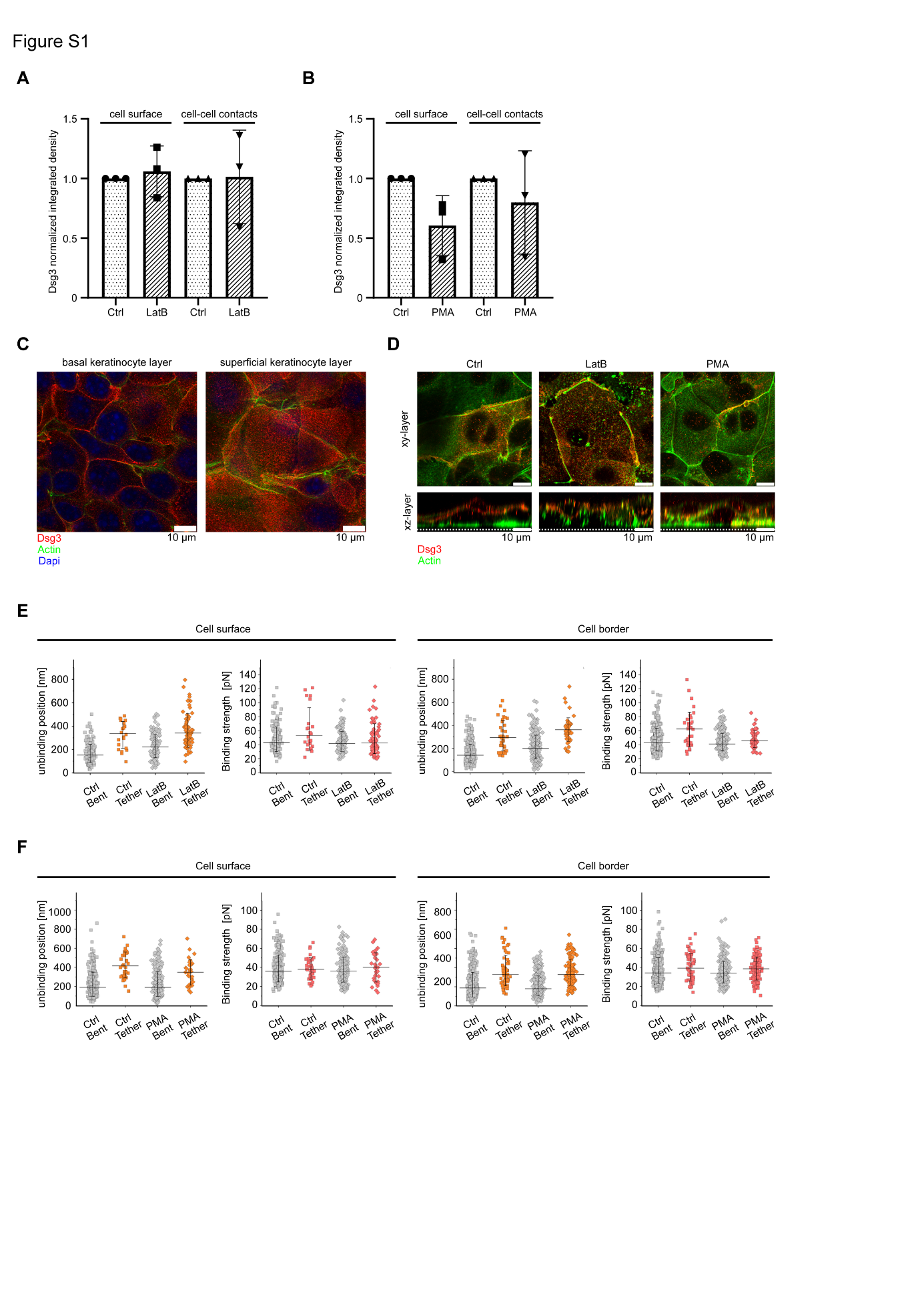

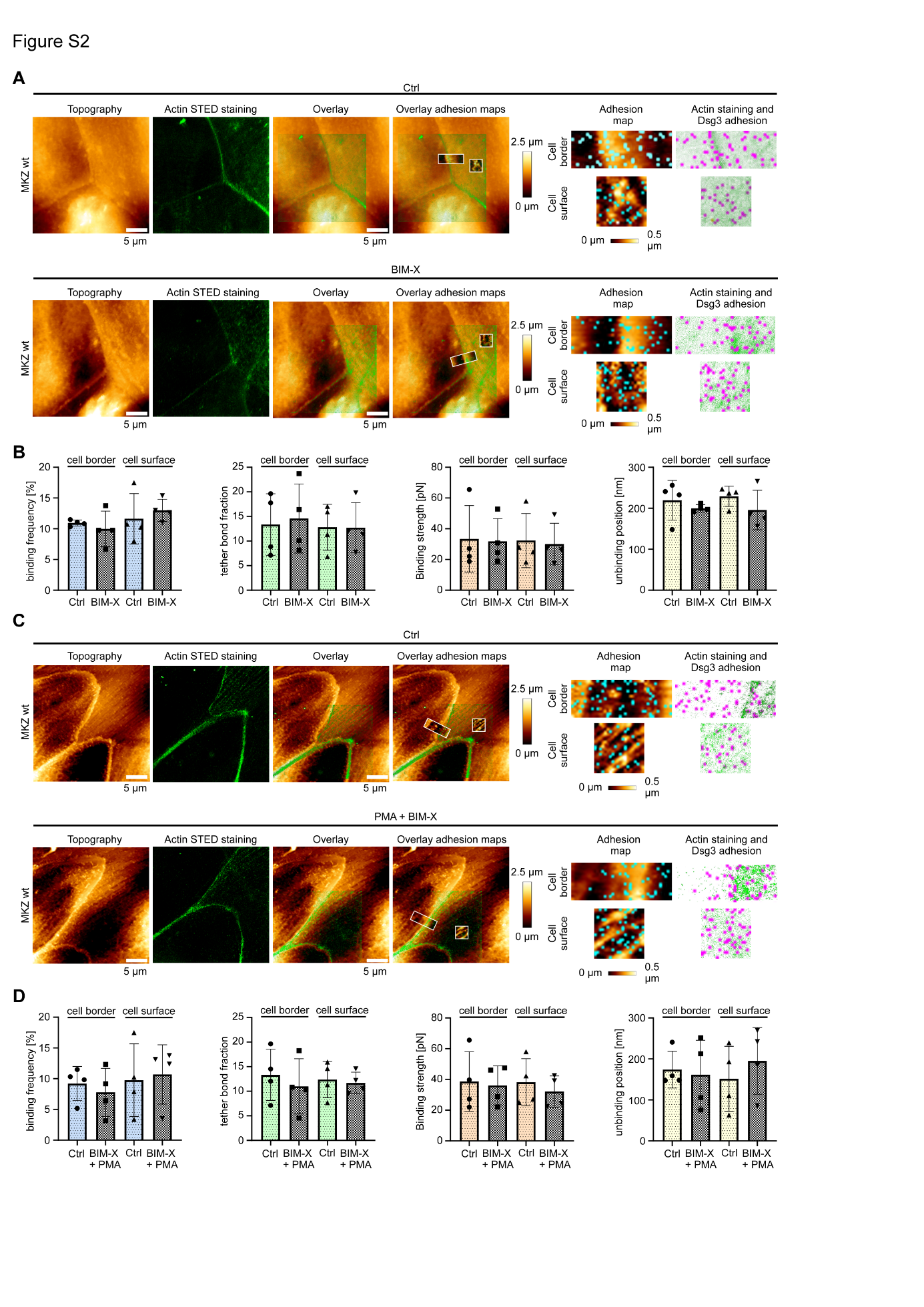

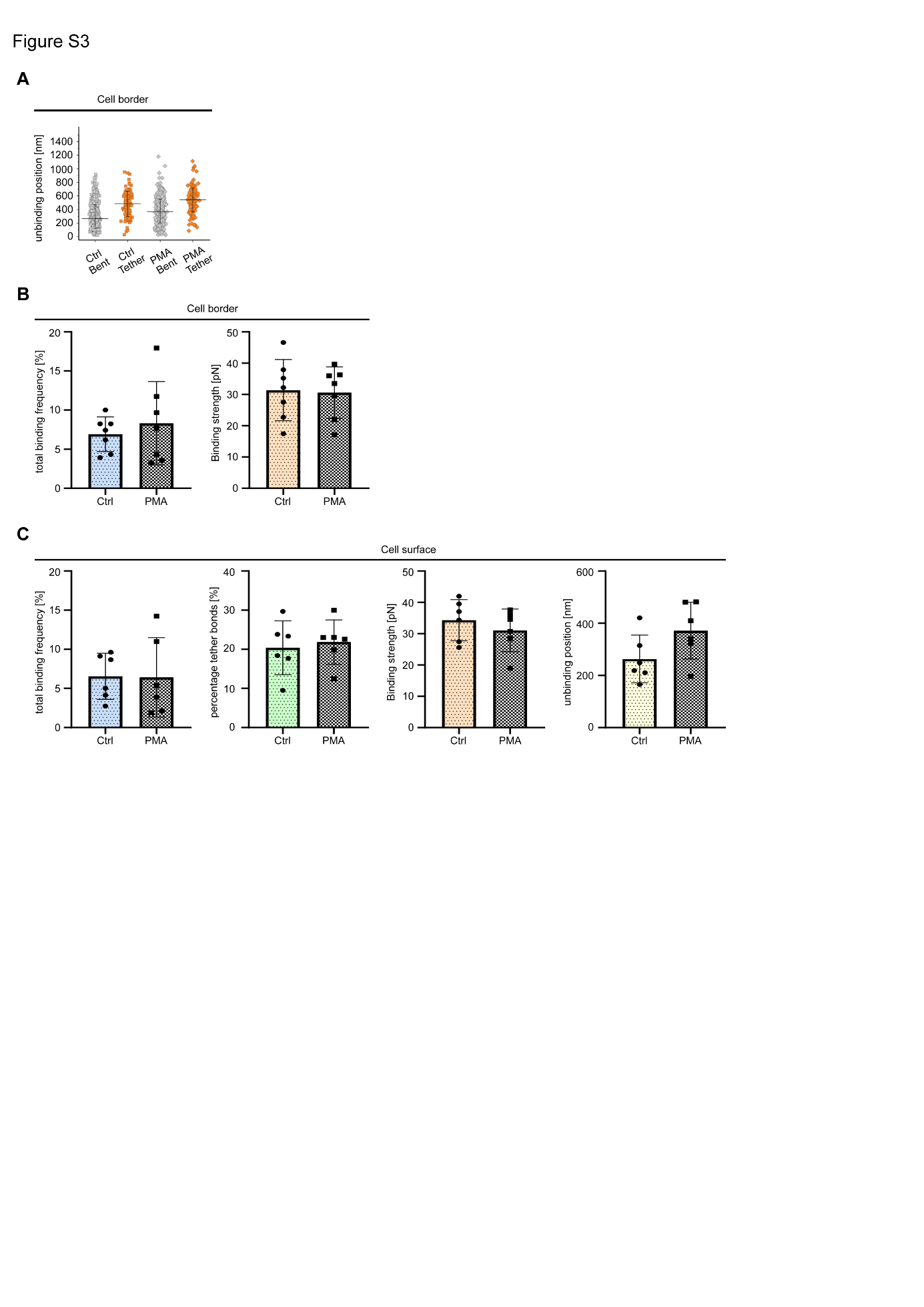

Supplement: Supplementary file 1 — Supplementary file1 (DOCX 2735 KB) [file 18_2022_4681_MOESM1_ESM.docx]
